# Policy recommendations for overdose response hotlines and applications as a harm reduction tool: A commentary

**DOI:** 10.17269/s41997-026-01180-3

**Published:** 2026-03-19

**Authors:** William Rioux, Nathan Rider, Jeremy Weleff, Boogyung Seo, Chelsea Shover, S. Monty Ghosh

**Affiliations:** 1 Department of Medicine, Faculty of Medicine & Dentistry, University of Alberta, Edmonton, AB Canada; 2 Department of Community Health Sciences, Cumming School of Medicine, University of Calgary, Calgary, AB Canada; 3 Department of Psychiatry, Faculty of Medicine & Dentistry, University of Alberta, Edmonton, AB Canada; 4 Neuroscience and Mental Health Institute, University of Alberta, Edmonton, AB Canada; 5 Department of Psychiatry, Yale School of Medicine, New Haven, CT USA; 6 Division of General Internal Medicine and Health Services Research, David Geffen School of Medicine at University of California Los Angeles, Los Angeles, CA USA; 7 Department of Internal Medicine, Faculty of Medicine & Dentistry, University of Alberta, Edmonton, AB Canada

**Keywords:** Opioid, Overdose, Harm reduction, Overdose response technologies, Policy, Opioïde, Surdose, Réduction des risques, Technologies d'intervention en cas de surdose, Politique

## Abstract

**Setting:**

The ongoing drug poisoning crisis continues to cause significant mortality, with a disproportionate number of overdose deaths occurring when individuals use drugs alone. While supervised consumption sites (SCS) have proven effective in reducing overdose fatalities, their impact is limited by geographic, social, and systemic barriers. In response, overdose response technologies have emerged to expand access to life-saving interventions beyond the reach of traditional harm reduction infrastructure.

**Intervention:**

Overdose response technologies (e.g., National Overdose Response Service (NORS)) and applications (e.g., Lifeguard App, UnityPhilly) offer real-time monitoring during solitary substance use. Hotlines provide peer-operated support and activate emergency responses if a caller becomes unresponsive, while apps use timers and geolocation to trigger automatic emergency services dispatch. Despite promising early outcomes, these services operate in a fragmented policy landscape without formalized regulatory guidance or implementation best practices.

**Outcomes:**

Preliminary data show that services like NORS have successfully prevented overdose deaths; however, published outcomes for most services remain limited. Key areas of priority for standards include the following: ensuring privacy for service, balancing data usage for quality improvement and research, building capacity to further equity of access to healthcare and harm reduction using the virtual platform, standardizing overdose response, and providing appropriate education around the efficacy of services.

**Implications:**

To enhance the effectiveness and sustainability of overdose response technologies, a comprehensive policy or standards framework is needed. This includes guidance on data privacy, service equity, public education, capacity-building, and outcome evaluation, laying the groundwork for safer, scalable, and more accessible overdose prevention interventions.

## Résumé

**Contexte:**

La crise actuelle liée à l'intoxication médicamenteuse continue d'entraîner une mortalité importante, avec un nombre disproportionné de décès par surdose chez les personnes solitaires qui consomment des drogues. Si les sites de consommation supervisée (SCS) se sont avérés efficaces pour réduire le nombre de décès par surdose, leur impact est limité par des obstacles géographiques, sociaux et systémiques. En réponse à cela, des technologies d'intervention en cas de surdose ont vu le jour afin d'élargir l'accès à des interventions vitales au-delà de la portée des infrastructures traditionnelles de réduction des risques.

**Intervention:**

Les technologies d'intervention en cas de surdose (par exemple, le National Overdose Response Service [NORS]) et les applications (par exemple, Lifeguard App, UnityPhilly) offrent une surveillance en temps réel pendant la consommation solitaire de substances. Les lignes d'assistance téléphonique fournissent un soutien assuré par des pairs et activent les services d'urgence si l'appelant ne répond plus, tandis que les applications utilisent des minuteries et la géolocalisation pour déclencher l'envoi automatique des services d'urgence. Malgré des résultats prometteurs, ces services fonctionnent dans un contexte politique fragmenté, sans directives réglementaires formelles ni de bonnes pratiques de mise en œuvre.

**Résultats:**

Les données préliminaires montrent que des services tels que le NORS ont permis de prévenir avec succès des décès par surdose; toutefois, les résultats publiés pour la plupart des services restent limités. Les principaux domaines prioritaires pour les normes sont les suivants: garantir la confidentialité du service; trouver un équilibre entre l'utilisation des données à des fins d'amélioration de la qualité et de recherche; renforcer les capacités afin de favoriser l'équité dans l'accès aux soins de santé et à la réduction des risques à l'aide de la plateforme virtuelle; standardiser les interventions en cas de surdose; et fournir une éducation appropriée sur l'efficacité des services.

**Implications:**

Afin d'améliorer l'efficacité et la durabilité des technologies d'intervention en cas de surdose, un cadre politique ou normatif complet est nécessaire. Celui-ci doit inclure des orientations sur la confidentialité des données, l'équité des services, l'éducation du public, le renforcement des capacités et l'évaluation des résultats, afin de jeter les bases des interventions de prévention des surdoses plus sûres, plus évolutives et plus accessibles.

## Introduction

The current opioid overdose crisis continues across North America and globally (Krausz et al., 2021). Strategies to lessen its impact will require a multifaceted public health strategy including prevention, harm reduction interventions, and treatment of substance use disorders (SUDs) (Humphreys et al., 2022; Livingston et al., 2022). Harm reduction programs, including supervised consumption sites (SCS), overdose prevention initiatives, naloxone distribution, and drug checking face challenges due to funding, “Not In My Backyard” (NIMBY)ism, and political will (Irvine et al., 2022; Rioux et al., 2023). Indeed, even in locations where SCS are established, their effectiveness is limited to a 500-m radius around these sites (Marshall et al., 2011) and most overdose deaths occur in individual’s own homes when using alone (Belzak & Halverson, 2018). Additional barriers to accessing SCS include fear of physical, sexual, or gendered violence, risk of criminalization, potential loss of employment or childcare, as well as personal preference to use at home (Rioux et al., 2023).

To address physical barriers to accessing SCS, various overdose response technologies including fixed devices, wearables, hotlines, and mobile applications have emerged (Rioux et al., 2023). These interventions appear to have been broadly overlooked in published strategies to address this crisis, despite emerging evidence for them as a tool to complement SCS. Nonetheless, a recent article by the Stanford-Lancet Commission has recommended technological solutions to address the aforementioned limitations of SCS (Humphreys et al., 2022; Livingston et al., 2022). Overdose response hotlines and applications (ORHA), including Canada’s National Overdose Response Service (NORS), and United States-based Never Use Alone, and SafeSpot programs connect callers to an operator who will enact a pre-agreed emergency response plan should the caller become unresponsive. Overdose response apps, including Alberta’s Digital Overdose Response Service, BC’s Lifeguard App, and Philadelphia’s Unity Philly, use timers and geolocation to trigger EMS dispatch automatically if a user does not respond to alarms.

In Canada, NORS has demonstrated the ability to successfully mitigate overdose events during solitary use and provide mental health and peer supports (Viste et al., 2023). Overdose response applications have also claimed to have saved lives; however, to date, only one service (Unity Philly) has peer-reviewed studies describing their outcomes (Schwartz et al., 2020). Outside of these two services, there is a stark paucity of literature on ORHA due to their novelty and diverse funding structures (such as program funding contingent on research grants and reports).

While brick-and-mortar SCS are the gold standard for supervised use, ORHA offers broader accessibility (Matskiv et al., 2022; Rioux et al., 2023). Nonetheless, the regulatory and policy environment within which ORHA operates remains largely undeveloped. As such, we propose key areas for standardization and policy/regulation, including enhancing data privacy while enabling data usage, building awareness of ORHA, standardizing overdose response, addressing barriers, and enabling outcome evaluation.

## A policy framework for overdose response hotlines and applications

### Enhancing data privacy while enabling data usage

While ORHA may provide additional anonymity than SCS (Seo et al., 2024), they require personal data such as location for EMS response. This creates potential legal and safety risks for clients, especially in jurisdictions where substance use remains criminalized (Byles et al., 2024). As such, it is vital to ensure that these services safeguard client privacy appropriately. Guaranteeing that appropriate data collection, use, protection, and disclosure standards are upheld is paramount to maintaining client trust in overdose response technologies (Seo et al., 2024). Currently, Canadian privacy laws such as the Personal Information Protection and Electronic Documents Act (PIPEDA) primarily apply to private commercial businesses or government institutions, leaving non-profit overdose services potentially underregulated (Canada 2014). By contrast, Europe’s General Data Protection Regulation (GDPR) applies universally. As such, governments could extend existing privacy frameworks to explicitly cover non-profit overdose response technologies. This could be achieved by creating a regulatory designation for ORHA services that mandates core safeguards (e.g., limiting data collection to what is necessary for EMS dispatch, requiring time-limited storage, and enforcing strict controls on secondary use), similar to GDPR’s “data minimization” and “purpose limitation” requirements. Such an approach would allow jurisdictions to protect client privacy while enabling the operational use of essential location data during emergencies.

### Evaluate and disseminate outcomes and research

Despite the importance of ensuring privacy for clients of ORHA, there is also a need to enable the use of data for quality improvement and research. This is doubly true as the political climate and public sentiment pertaining to harm reduction services and SCS, in particular, appear to be shifting, with reports of questionable methodology being used to justify their closure (Salvalaggio et al., 2023). Governments should work in partnership with ORHA and service users to support high-quality research on their impacts and effectiveness. Currently, few services, other than NORS and Unity Philly, are represented in peer-reviewed literature (Matskiv et al., 2022; Schwartz et al., 2020; Viste et al., 2023). Furthermore, most research on overdose response hotlines and applications has been qualitative (Rioux et al., 2023). In our view, there is clearly a need for improved evaluation of existing and emerging ORHA. In contrast, brick-and-mortar harm reduction programs like SCS in Canada continuously provide evaluation metrics in conjunction with funding mandates from respective national and provincial governments and are widely evaluated in the literature (Caulkins et al., 2019; Humphreys et al., 2022). Governments should insist on transparency on behalf of service operators, which may help ensure the safety of people who use drugs and support making more informed decisions regarding the harm reduction technologies and services they use. 

Governments should require reporting on service effectiveness while protecting client privacy and should be co-designed with people with lived/living experience to ensure relevance (Michaud et al., 2022). A recent multi-round Delphi study conducted with people with lived and living experience of substance use, ORHA service operators, and researchers has highlighted key demographic and service use indicators for evaluating these services (Rioux et al., 2025). While policy makers were not included within this study, metrics including neighbourhood, age, gender, past overdose experience, substance used, amount and route of use, and service use outcomes including response type, morbidity and mortality, overdose events, responder arrival time, and post-rescue care were recommended to balance client privacy, service evaluation and staffing burdens (Rioux et al., 2025). Similarly, the aforementioned suggestions represent minimum safeguards for client safety and evaluation of service efficacy but must nevertheless be context-sensitive, population-specific, and adaptable to local needs, dynamics, and contexts.

Even without research and evaluation, it is imperative that some information on outcomes from the various services is shared with the public as well as with public health regulators, as transparency of outcomes and standardization of reported metrics can build public trust and foster policy alignment.

### Delivery and operational standardization

At present, no frameworks detail critical service components and operational aspects or regulations for ORHA despite variation in service scope (Rider et al., 2023). Some services offer mental health support, stimulant-induced psychosis de-escalation, or referrals to social services (Rider et al., 2023). Given concerns regarding mortality risks due to delayed response times relative to the gold standard of SCS (Rioux et al., 2023), decision-makers may wish to require that users be informed of these risks when using hotlines and applications for overdose prevention or consider standard response time expectations. Policymakers should develop distinct frameworks for hotline and app-based services, addressing referral pathways, operational requirements, and expected EMS response times.

Furthermore, staff training is essential, particularly for those who frequently work with vulnerable and marginalized populations (Canadian Center on Substance Use & Addiction, 2020). Training should incorporate harm reduction and trauma-informed principles. Equally important are supports for peer operators, given the risk of burnout and compassion fatigue (Seo et al., 2024).

Lastly, given the unique operational structure of ORHA and the inherent delay in response times innate to utilizing emergency medical services (Mell et al., 2017), governments should work with services and EMS to develop response standards specific to these services. Such standards might include minimum expected response times and expectations for when emergency or peer response should be initiated or for disconnected calls and location failures. This further highlights the importance of shared decision-making and education surrounding the risks related to delayed response, such as those that may be seen in rural communities.

### Supporting equitable access to harm reduction

ORHA can help overcome current inequities in access to harm reduction services, particularly for rural, remote, and gender diverse populations (Perri et al., 2022; Rioux et al., 2023). Legal rulings have limited SCS expansion in the United States, however, ORHA such as “SafeSpot”, as well as one application, Philadelphia’s “Unity Philly”, can fill critical gaps. Furthermore, many low- and middle-income countries currently lack harm reduction programs despite high needs, and these services may provide a less costly, more accessible option for some nations (O’Keefe et al., 2019).

In countries in which SCS are established, they are often concentrated in urban areas due to infrastructure costs, creating regional disparities (Mocanu et al., 2024). As such, the proliferation of ORHA can reduce these barriers, though challenges including poor emergency response times in remote areas remain (Mell et al., 2017). Governments should consider not only how to offer ORHA to areas that are underserved by existing harm reduction services, but also how to integrate these services with existing health, social, and harm reduction services as seamlessly as possible to reduce the burden of service navigation. Indeed, ensuring that both health and harm reduction-related services are not only aware but supportive of these types of interventions is instrumental to their success, particularly during emergency response situations. While ORHAs are, in our view, part of the key to expanding harm reduction access to populations whose access was previously limited, we recognize that they are not sufficient alone and present their own barriers to access, including requiring clients to have access to a working phone, internet or WiFi, or data plan (Rioux et al., 2023). Furthermore, we acknowledge that hotlines and applications may best serve housed individuals compared to individuals experiencing homelessness. As such, a multifaceted approach including both in-person and virtual harm reduction programming would likely result in the best outcomes.

Additional notable barriers to accessing brick-and-mortar SCS or overdose prevention sites are limited hours of operation and lack of support for individuals’ preferred route of administration (such as safer smoking sites) (Mocanu et al., 2024). Indeed, one study of clients of an overdose prevention hotline found that 17.5% of those who accessed the service did so while their neighbourhood supervised consumption site was closed, and 12.4% did so due to a lack of support for their preferred route of administration of smoking (Mocanu et al., 2024). Consequently, policy approaches to ORHA should incentivize the provision of 24-h access and support all routes of administration to fill critical gaps in harm reduction service access.

### Facilitating awareness and education

The potential reach and uptake of ORHA are substantial, particularly within underresourced and equity-deserving communities (Mocanu et al., 2024; Rioux et al., 2023); however, overall uptake remains low compared to the total size of the target population who may benefit from these services (Viste et al., 2023). Consequently, education campaigns should target high-risk populations, including those discharged from hospitals or released from incarceration (Gunn et al., 2018; Keen et al., 2021). Current public health programs often provide naloxone to these at-risk groups (Gunn et al., 2018), and including messaging about ORHA may help to facilitate uptake (Safi et al., 2023). Education must also address community misconceptions about overdose technologies. Public messaging like “never use alone” or “carry naloxone” can normalize safer practices. Governments should support local partnerships with healthcare, housing, and social services to integrate overdose response tools into existing care pathways.

### Building capacity for sustained operation

Governments must ensure that services can scale with increased awareness. Stable funding is critical so that organizations can focus on quality and research rather than pursuing uncertain funding.

Furthermore, governments should avoid creating overly burdensome requirements for the integration of ORHA within communities, such as those that currently exist for many SCS (Canadian Center on Substance Use & Addiction, 2020). Such regulatory hoops are, in our view, less necessary for ORHA, which offers distributed services to people who are already using substances in their community. Nonetheless, governments should still hold these services to a high standard of care and professionalism to facilitate the best possible service delivery on par with other health resources and community-based health service provision.

### Addressing barriers to use

Police involvement in overdose responses remains a major deterrent to using these services, despite Good Samaritan Laws (van der Meulen et al., 2021). These laws often have limited scope and do not prevent police from attending overdose events, which can retraumatize individuals or lead to collateral consequences (Michaud et al., 2025; van der Meulen & Chu, 2022). Consequently, some individuals may choose not to access emergency medical services or ORHA, resulting in potentially fatal consequences (Seo et al., 2024). Despite this, it is important to note that police responses have accelerated the time taken to administer naloxone and, at times, may be the only form of overdose response in some communities (Pourtaher et al., 2022). As such, government organizations that have already implemented such Good Samaritan Overdose Acts should consider further reducing police callouts to overdoses where possible and without reducing the quality of care (van der Meulen et al., 2021) to facilitate 911 calls for overdose and eliminate a barrier to the use of overdose response technologies.

Services should also reflect cultural and regional contexts. Co-design of ORHA with people who use substances should be paramount (Murphy et al., 2024). While there are a few examples of incorporating these views into current overdose applications, such as the Lifeguard app’s collaboration with the Metis Nation of Canada (*Métis Nation BC Launches Life-Saving Technology* 2021), continued work in this area will help to improve cultural relevance and may improve equity of service access overall.

### Implications for policy and practice

Overdose response hotlines and applications represent an evolving frontier in harm reduction policy and practice. As these services gain traction, they must be integrated into broader public health and healthcare systems through thoughtful regulation, sustainable funding, and cross-sector collaboration while remaining adaptable to changing drug markets and local contexts. Policymakers should recognize these tools as complementary interventions alongside physical SCS and other harm reduction services. To maximize impact, governments must prioritize equitable access by expanding these services into rural, remote, and underserved communities, and by addressing technological barriers such as phone, data, and internet access.

Service standards should be developed collaboratively with people who use drugs, emphasizing trauma-informed and culturally safe care, while maintaining flexibility to support peer-led and grassroots models. Data governance frameworks are urgently needed to ensure client privacy while enabling quality improvement and service evaluation. Embedding overdose response services into existing health and social care networks, including primary care, addiction services, housing, and emergency response, will strengthen continuity of care and foster trust among both users and providers.

Importantly, policy must address systemic barriers to use, such as criminalization and fear of police involvement, by refining Good Samaritan legislation and reducing unnecessary law enforcement attendance at overdose events. Public awareness campaigns and education for healthcare workers, first responders, and community members can further normalize and destigmatize these services. Ultimately, policy and practice must evolve in parallel to support overdose response technologies as part of a comprehensive, evidence-based, and compassionate response to the ongoing drug poisoning crisis.

## Conclusions

In conclusion, this manuscript highlights key areas of policy and implementation improvement that may increase the effectiveness, acceptability, and uptake of ORHA. Governments should prioritize privacy for service clients while enabling data usage for quality improvement and research. Furthermore, it is critical to build capacity to further equity objectives by reaching underserved client populations. Increased standardization of services and the development of service standards will enhance the client experience and improve safety while facilitating appropriate education of clients and the public alike. Finally, governments should address barriers to the use of overdose response hotlines and applications while recognizing that these novel technologies are only part of a necessary suite of harm reduction approaches.

## Data Availability

This manuscript is a commentary and does not report original empirical data. As such, no datasets were generated or analyzed, and data sharing does not apply.
